# Supplementary material for: Immunoglobulin expression and the humoral immune response is regulated by the non-canonical poly(A) polymerase TENT5C
Source: Nat Commun. 2020 Apr 27;11:2032. doi: 10.1038/s41467-020-15835-3 (PMC7184606; doi:10.1038/s41467-020-15835-3)
Supplement: Supplementary file 1 — Supplementary Information [file 41467_2020_15835_MOESM1_ESM.pdf]

Supplementary Information

Immunoglobulin expression and the humoral immune response is regulated by the noncanonical poly(A) polymerase TENT5C

Bilska et al.

Supplementary Figures 1 - 8

Supplementary Tables 1 - 7

Supplementary References

Supplementary Figure 1

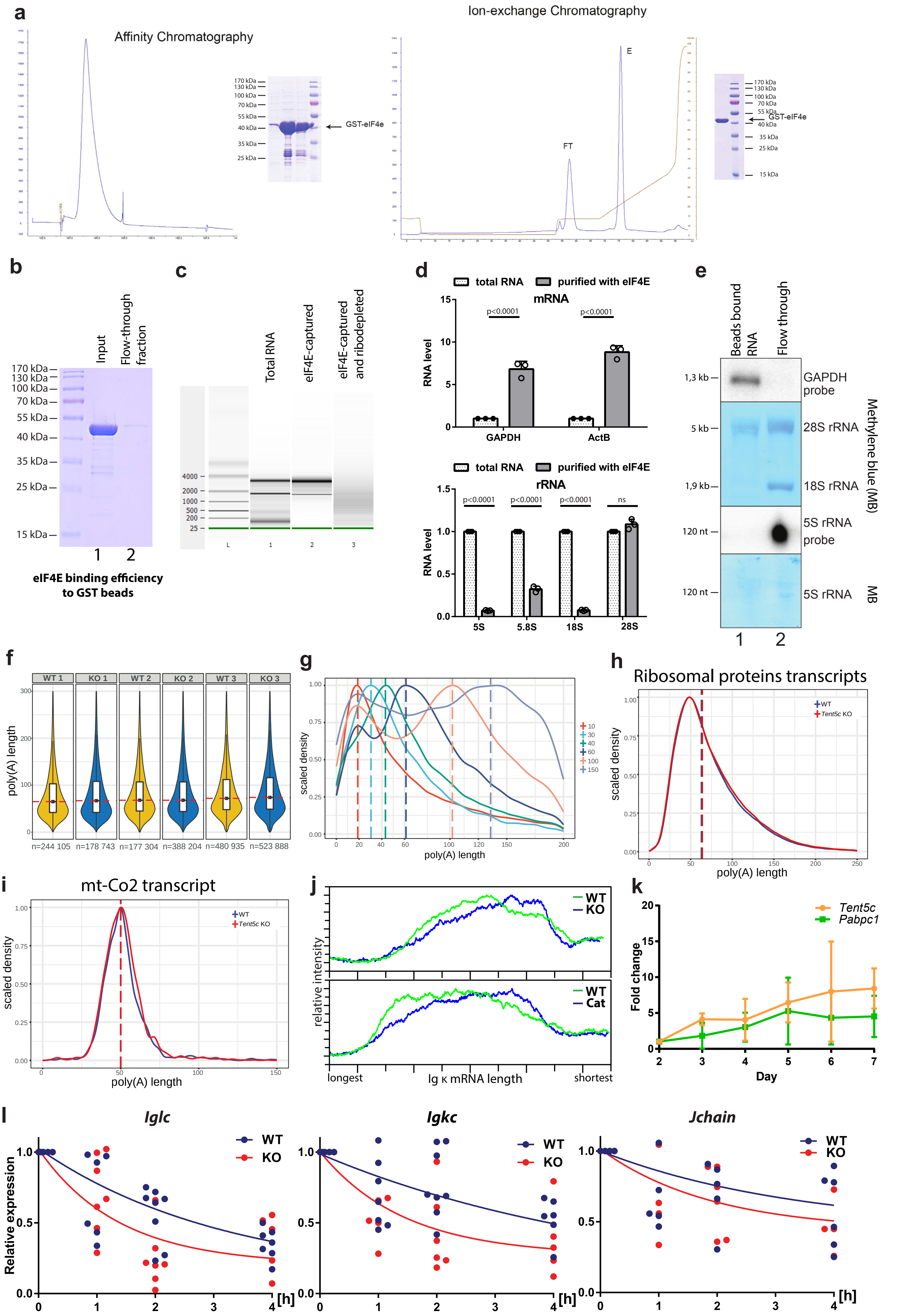

Supplementary Figure 1 (Related to Fig. 1). Direct RNA Sequencing is a reliable method to measure poly(A) tails in responding B cells.

a, Purification of the GST-eIF4E protein. Affinity chromatography and ion-exchange chromatography profiles with SDS-PAGE analysis of the purified GST-eIF4E protein.

b, Validation of the purified eIF4E batch binding to GST beads. Coomassie Blue-stained SDSPAGE gel analysis of purified eIF4E input and a flow-through fraction after incubation with GST beads.

c, Agilent 2100 Bioanalyzer electropherogram profiles of total RNA, eIF4E-captured RNA and both eIF4E-captured and ribodepleted RNA from WT B cells activated for 7 days.

d, The efficiency of mRNA enrichment (higher panel) and rRNA removal (lower panel) after eIF4E capture assessed by qPCR analysis. Total RNA from HEK293T cells were purified with eIF4E protein. Then input and purified RNA were subjected to qPCR. Bars represent mean values
±SD (*n*=3 technical repeats), P values calculated using the two-tailed unpaired Student's t-test are indicated on figure; ns – not significant.

e, Northern blot analysis of eIF4E-capture efficiency. Total RNA from HEK293T cells were purified with eIF4E protein. Then purified RNA and unbound flow-through fraction were compared.

f, Comparison of global poly(A) tail lengths distribution in all individual Nanopore sequencing runs. Red dashed lines represent median poly(A) length for each condition.

g, Poly(A) lengths estimation results for control eGFP transcripts with predefined poly(A) tails length of 10, 30, 40, 60, 100 and 150 nt. Vertical dashed lines indicate the most frequently observed poly(A) length for each spike-in.

h-i, Nanopore-based poly(A) lengths profiling of B cells isolated from WT and *Tent5c* KO, activated 4 for 7 days. Shown are density distribution plots, scaled to a maximum of 1, for (**h**) ribosomal proteins transcripts, (**i**) mitochondrially encoded cytochrome c oxidase II transcript. Vertical dashed lines represent median poly(A) lengths for each condition **j,** Densitometry plots of the northern blots shown in Figure 1h (lanes 1 and 2) and Figure 8a (lanes 1 and 2).

k, qPCR analysis of *Tent5c* and *Pabpc1* expression levels in WT B cells activated for 2-7 days.
Dots represent mean fold change values ±SD (*n*=4, biological replicates). Pearson's correlation between *Tent5c* and *Pabpc1* expression r=0.88.

l, RNA half-life analysis after actinomycin-D treatment. WT and *Tent5C* KO B cells activated 3 days were treated with actinomycin-D up to 4h and decrease of mRNAs encoding J chain and κ and λ light chains immunoglobulins levels were estimated by RT-qPCR analysis (values are shown as fold changes normalized to time 0; *n*=6-8, biological replicates). The curves were obtained by nonlinear regression (one phase decay analysis).

Source data are provided as Supplementary Figure 8 (1a,b,e), as a Source Data file (1d,k,l) and in Supplementary Dataset 1 (1f-i).

Supplementary Figure 2

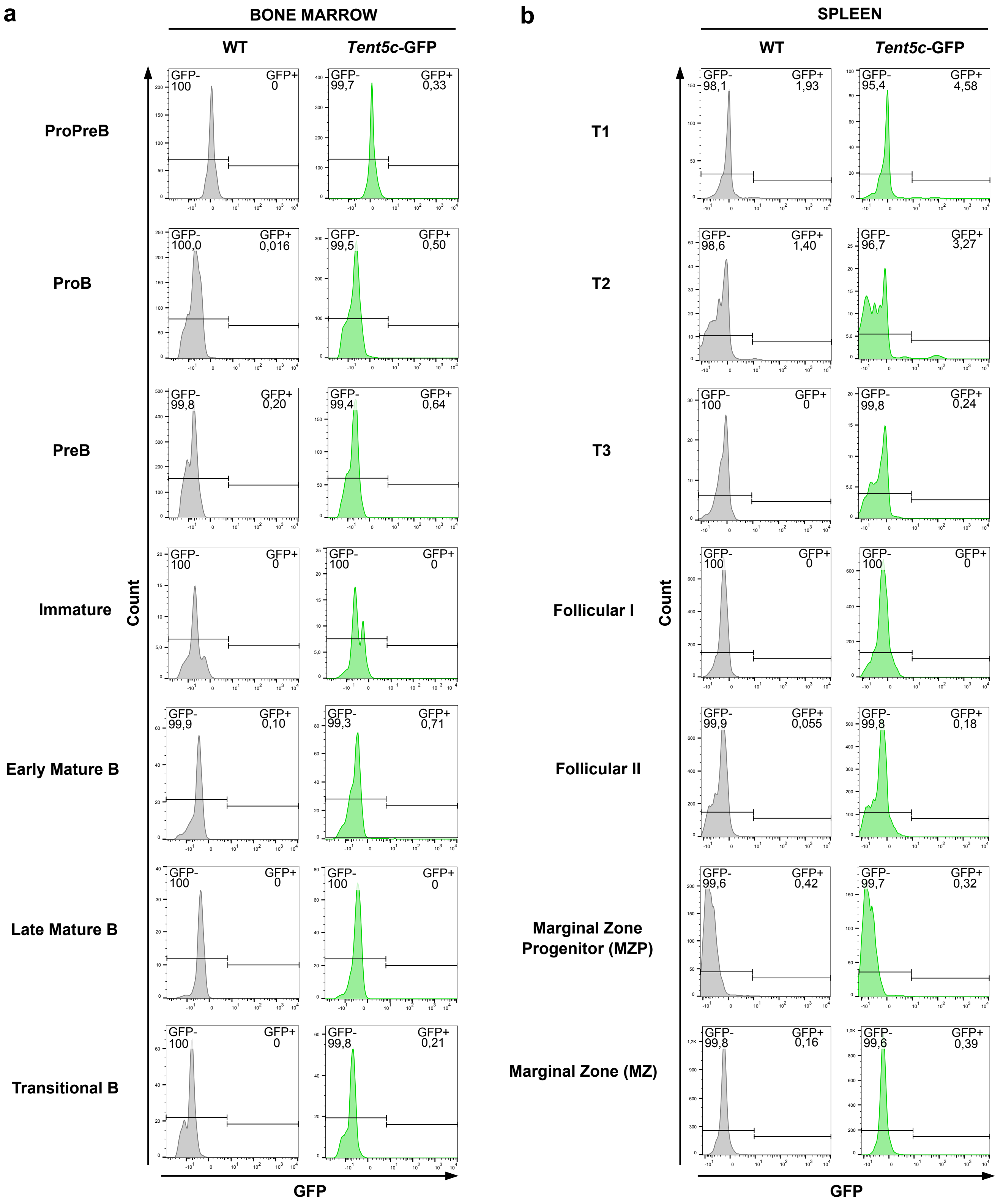

Supplementary Figure 2 (Related to Fig. 3). Flow cytometry analysis of TENT5C-GFP expression in B cells subpopulations.

a-b, Histograms showing GFP fluorescence intensity in different B lymphocytes subsets from WT or *Tent5c*-GFP bone marrow (**a**) and spleen (**b**). Cells from bone marrow were stained based on the CD19, CD43, CD45R, CD24, CD249, IgM and IgD markers. Cells from spleen were stained based on the CD19, CD23, CD93, CD45R, CD21, IgM and IgD markers. Staining panels distinguish following B cell subsets: ProPreB, ProB, PreB, Immature, Early Mature B, Late Mature B, Transitional B, T1/T2/T3, Follicular I and II, Marginal Zone Progenitors and Marginal Zone. See also the gating strategy in Supplementary Figure 3.

Supplementary Figure 3

a

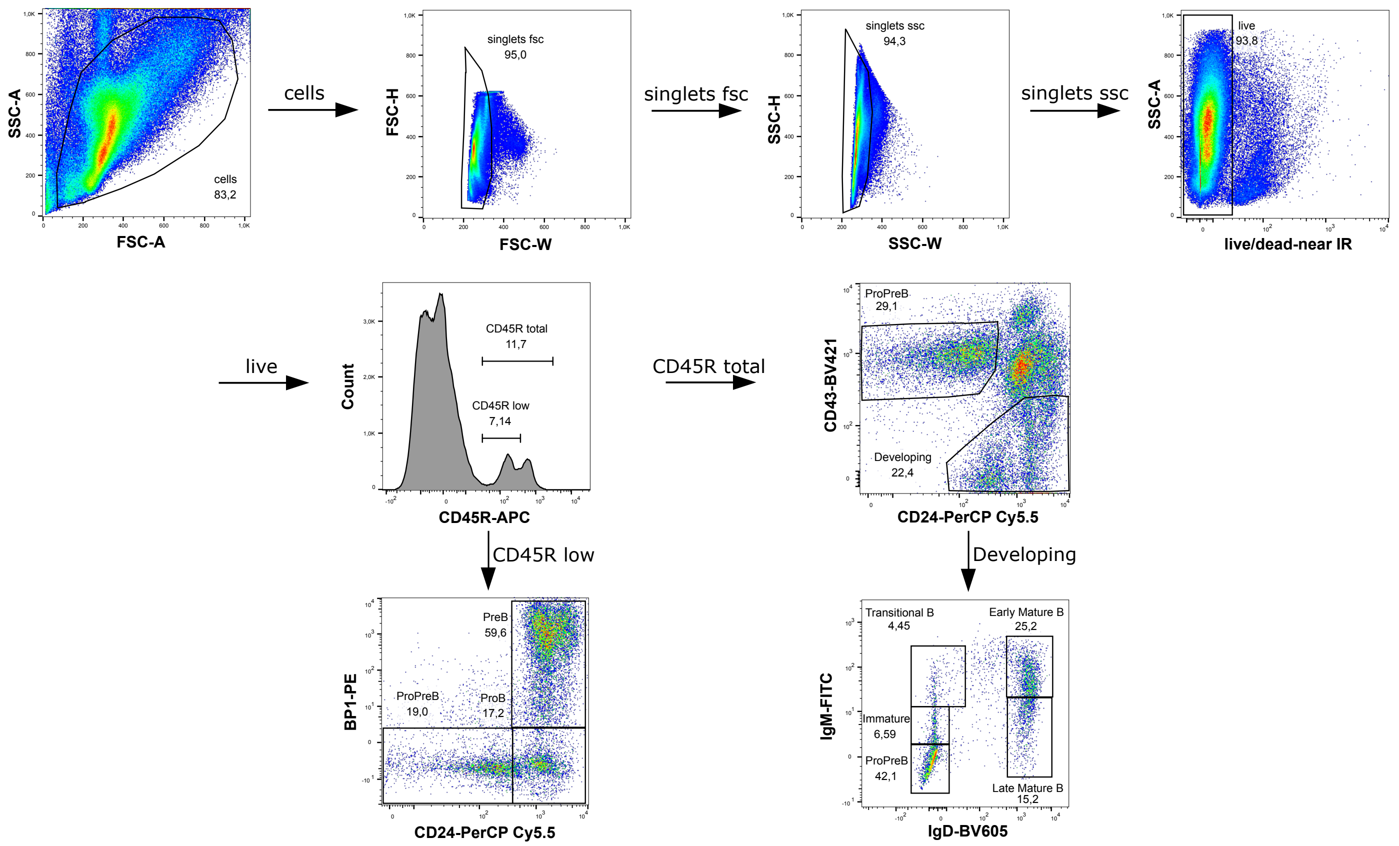

b

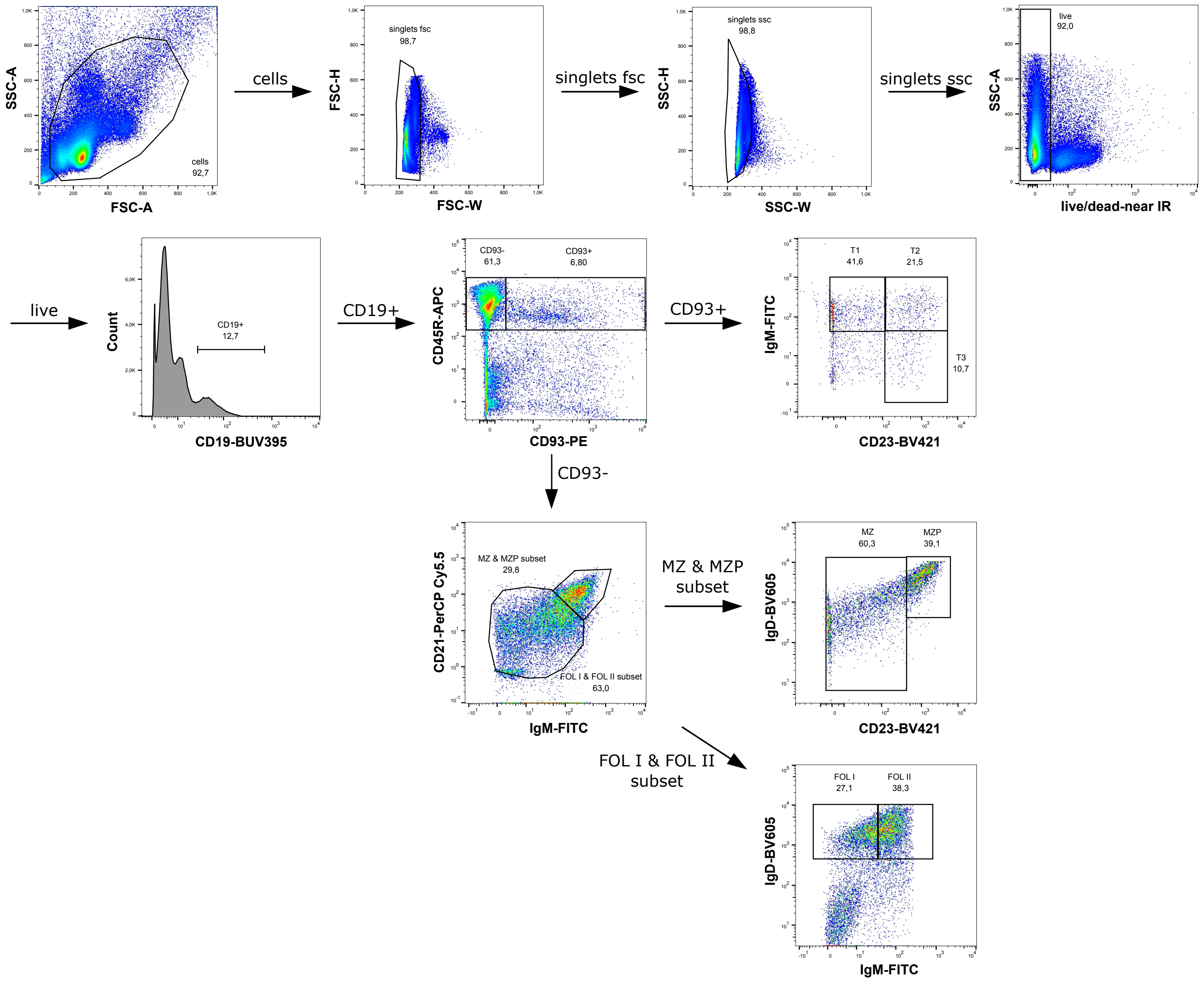

Supplementary Figure 3 (Related to Figure 2, Supplementary Figure 2, Supplementary Figure 6 and Supplementary Figure 7). Gating strategy of bone marrow B cells staining panel (a) and spleen B cells staining panel (b).

a, The 8-colour flow cytometry analysis of B cells isolated from bone marrow. Cells were stained with fluorochrome-conjugated antibodies against CD19-BUV395, CD43-BV412, IgM-FITC, CD249-PE, IgD-BV605, CD45R-APC, CD24-PerCP Cy5.5. When the TENT5C-GFP level was checked, antibody against IgM-BV510 was used. To exclude dead cells LIVE/DEAD™ Fixable Near-IR Dead Cell Stain Kit was used.

b, The 8-colour flow cytometry analysis of B cells isolated from spleen, Cells were stained with fluorochrome-conjugated antibodies against CD19-BUV395, CD23-BV421, IgM-FITC, CD93-PE, IgD-BV605, CD45R-APC, CD21-PerCP Cy5.5. When the TENT5C-GFP level was checked, antibody against IgM-BV510 was used. To exclude dead cells LIVE/DEAD™ Fixable Near-IR Dead Cell Stain Kit was used.

Supplementary figure 4

Gating strategy of plasmacytes staining panel

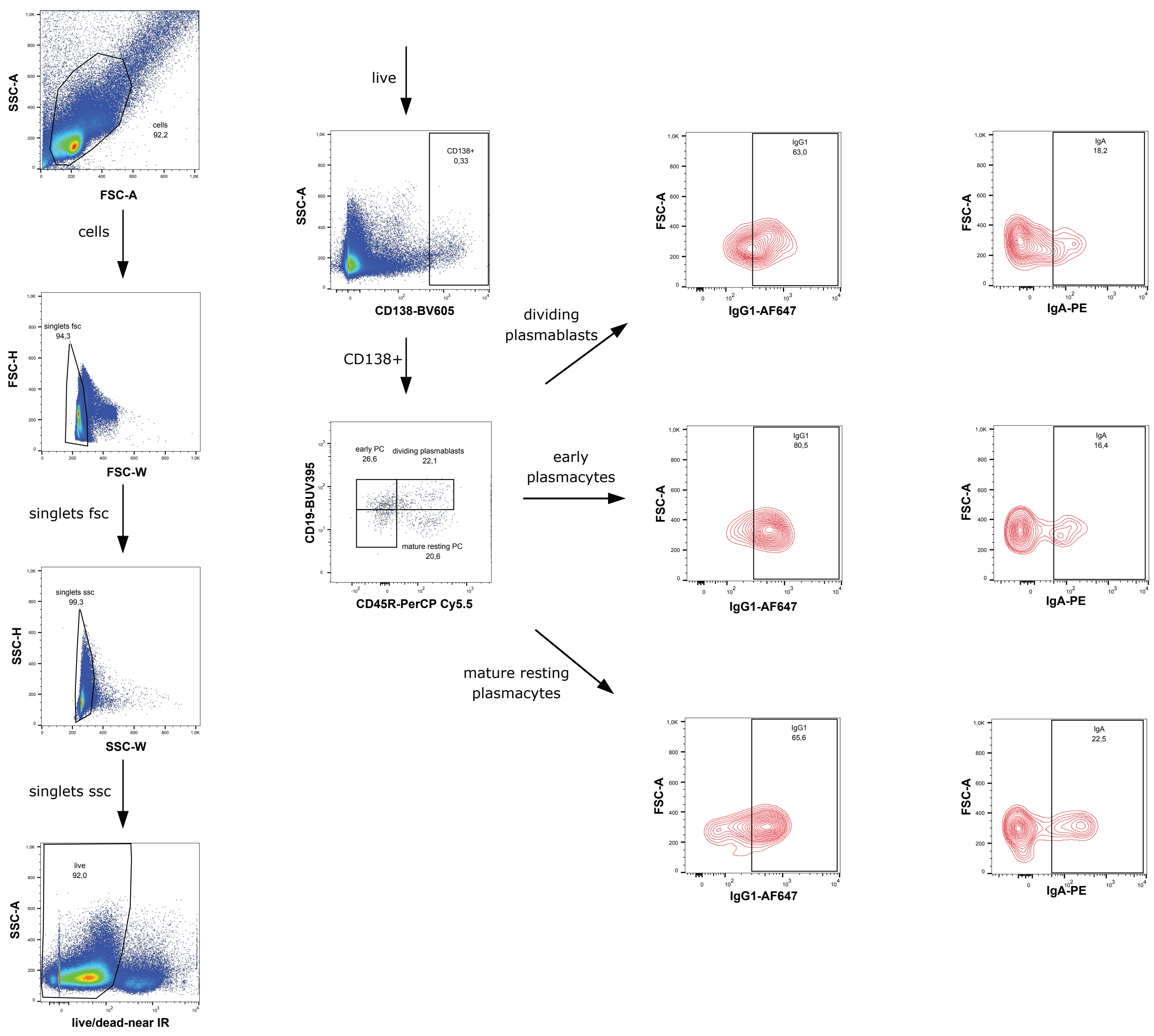

Supplementary Figure 4 (Related to Figure 2, 3, 4, 5, 6, 7, 8). Gating strategy of plasmocytes staining panel.

A 7-colour flow cytometry analysis of plasma cells isolated from bone marrow or spleen. Cells were stained with fluorochrome-conjugated antibodies against CD19-BUV-395, IgM-FITC, IgA-PE, CD138BV605, IgG1-AF647, CD45R-PerCP Cy5.5 (see analysis on Fig. 5. 6 and 7). For intracellular (cytoplasmic) immunoglobulins measurement (see Fig. 2) the same gating strategy was applied. When the TEN5C-GFP level was analysed antibody against CD-138-PE and CD-AF700 was used (Fig. 3), for measuring ER, the ER tracker-Red was used (Fig. 7). To exclude dead cells LIVE/DEAD™ Fixable Near-IR or Violet Dead Cell Stain Kit was used.

Supplementary Figure 5

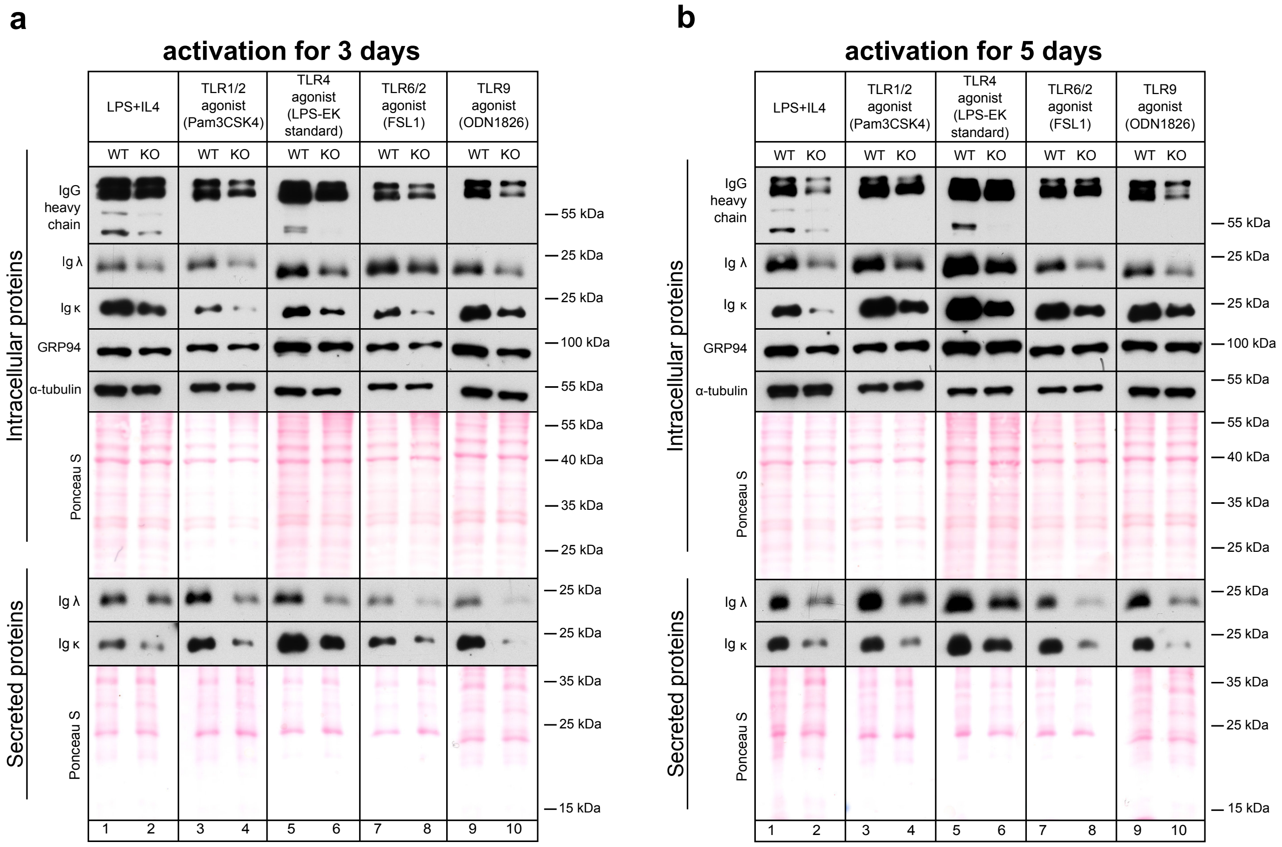

Supplementary Figure 5 (Related to Fig. 4). *Tent5c* KO B cells activated by innate signaling pathways produce fewer antibodies.

a,b, Western blot analysis of IgG heavy and light chains (λ and κ), intracellular and secreted level, in *Tent5c* KO and WT B cells, activated with TLR agonists: LPS/IL-4 (positive control; lanes 1,2), TLR 1/2 (lanes 3,4), TLR4 (lanes 5,6), TLR6/2 (lanes 7,8) and TLR9 (lanes 9,10) for 3 (**a**) or 5 (**b**) days. GRP94 was used as an activation marker, α-tubulin and Ponceau S staining were used as loading controls. Source data are provided as Supplementary Figure 8i,j.

Supplementary Figure 6

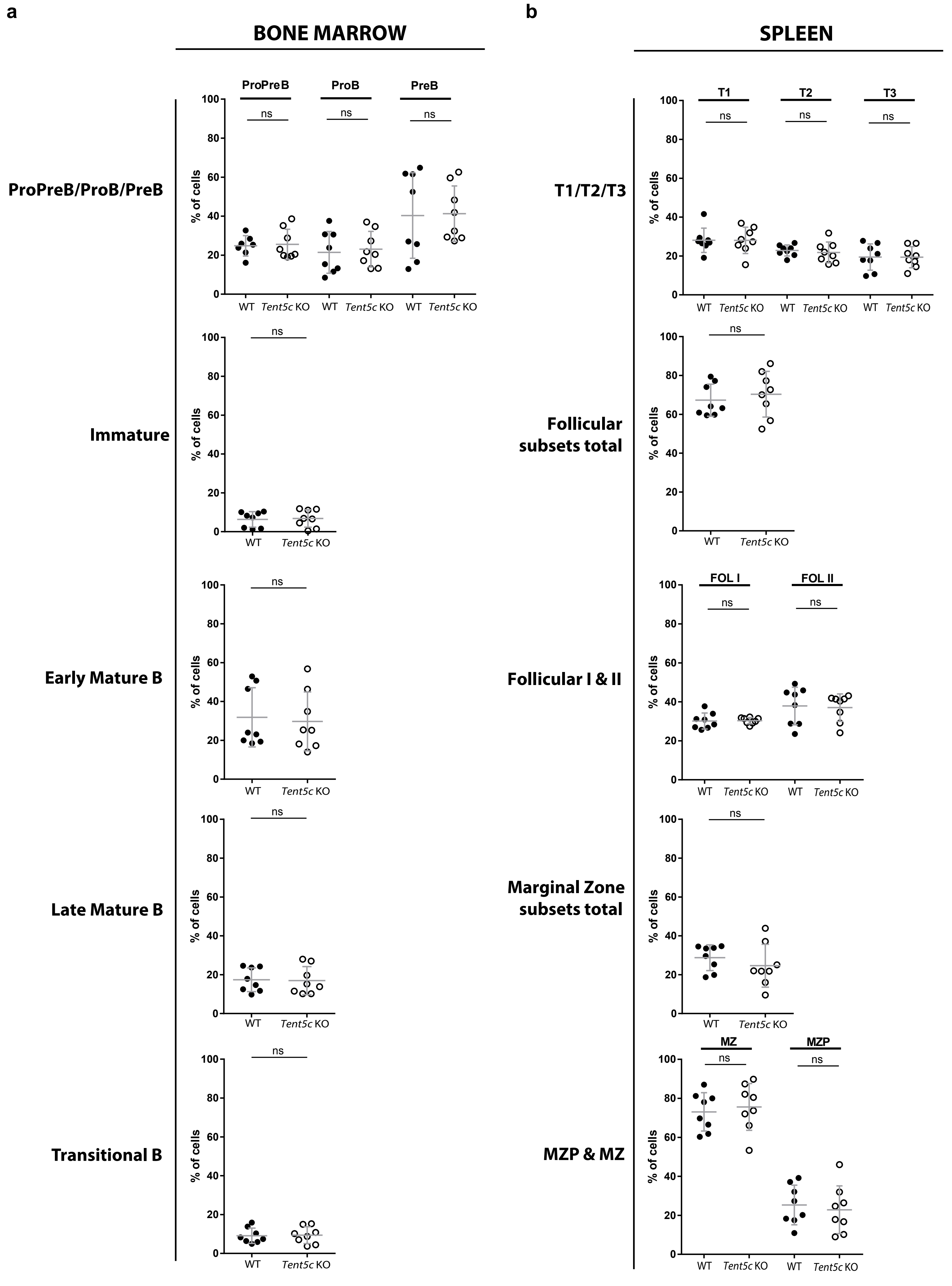

Supplementary Figure 6 (Related to Fig. 5). TENT5C expression does not influence the early stages of B cells development.

a,b, Comparison of different B cells subsets isolated from WT or *Tent5c* KO from bone marrow (**a**) and spleen (**b**) based on the flow cytometry results. Subpopulations of ProPreB, ProB, ProB, Immature, Early Mature B, Late Mature B, Transitional B, T1/T2/T3, Follicular I & II, Marginal Zone Progenitors (MZP) and Marginal Zone (MZ) were checked based on CD19, CD43, CD45R, CD24, CD249, IgM, IgD, CD23, CD93, CD21 markers. See the gating strategy in Supplementary Figure 3. Data are shown as a mean of WT n=8, *Tent5c* KO n=8 mice, two-way ANOVA with Bonferroni post hoc test. Source data are provided as a Source Data file.

Supplementary Figure 7

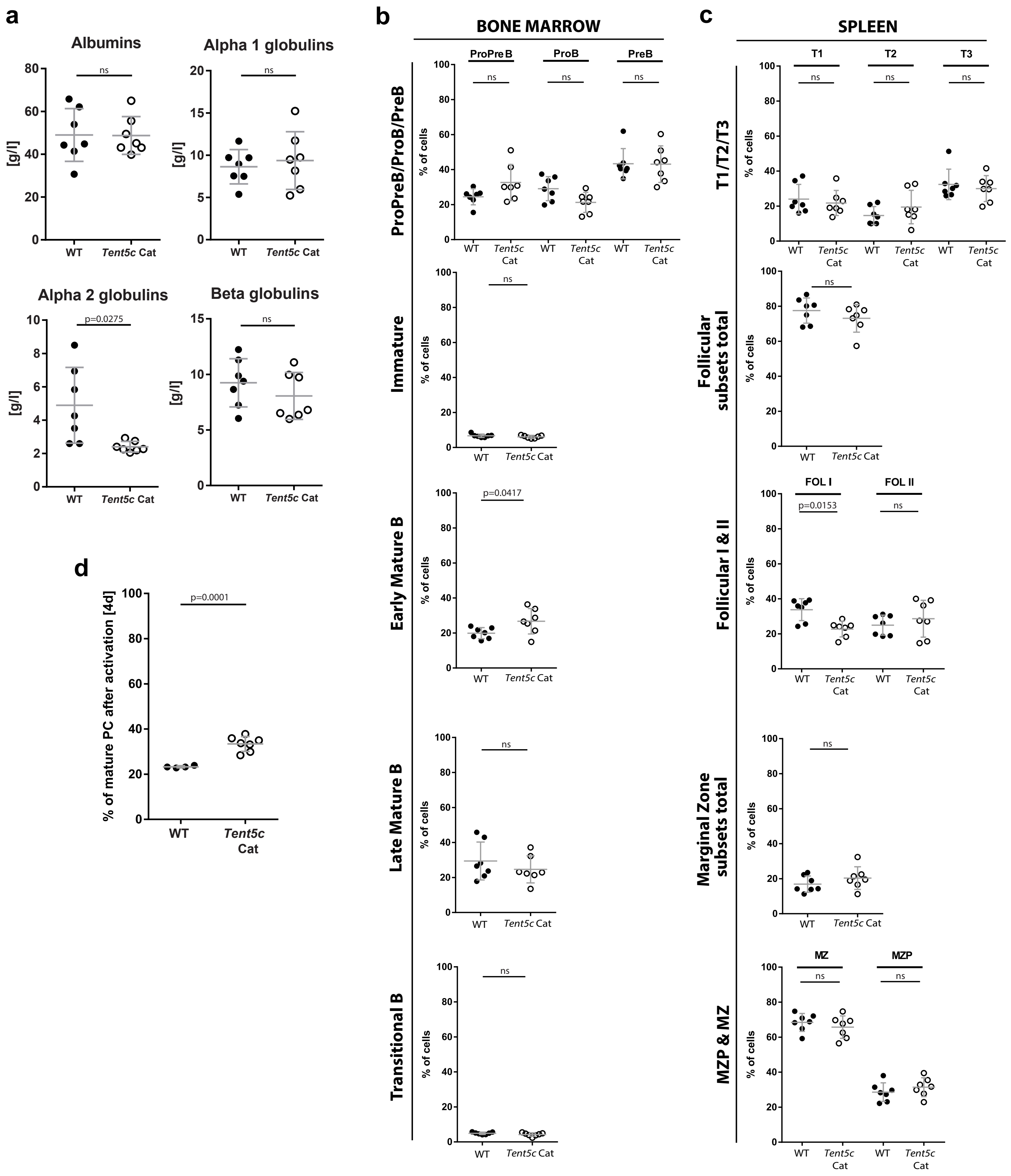

Supplementary Figure 7 (Related to Fig. 8). TENT5C expression does not influence the early stages of B cells development.

a, Examination of blood serum albumins, alpha 1 globulins, alpha 2 globulins, and beta globulins levels in *Tent5c* Cat and control animals by SPEP (*n*=7).

b,c, Comparison of different B cells subsets isolated from WT or *Tent5c* Cat from bone marrow (b) and spleen (c) based on the flow cytometry results. Subpopulations of ProPreB, ProB, ProB, Immature, Early Mature B, Late Mature B, Transitional B, T1/T2/T3, Follicular I & II, Marginal Zone Progenitors (MZP) and Marginal Zone (MZ) were checked based on CD19, CD43, CD45R, CD24, CD249, IgM, IgD, CD23, CD93, CD21 markers. The gating strategy is shown in Supplementary Figure 3 (WT *n*=7, Cat *n*=7)

d, Percentage of mature PC positive cells in bone marrow and spleen (WT *n*=4, Cat *n*=7).

Two-tailed unpaired Studen's t-test with Welch's correction (**a**, **d**), two-way ANOVA with Bonferroni post hoc test (**b**, **c**, concerns analysis of PreProB/ProB/PreB, T1/T2/T3, Follicular I & II and MZP & Mz of), two-tailed unpaired Student's t-test (**b**, **c** – other panels); not significant (ns); data are presented as mean values ±SD; *n* – biological replicates.

Source data are provided as a Source Data file.

Supplementary Figure 8 related to Figure 1h, 2a, 2b, 5a and 8a.

a

Figure 1 and 8

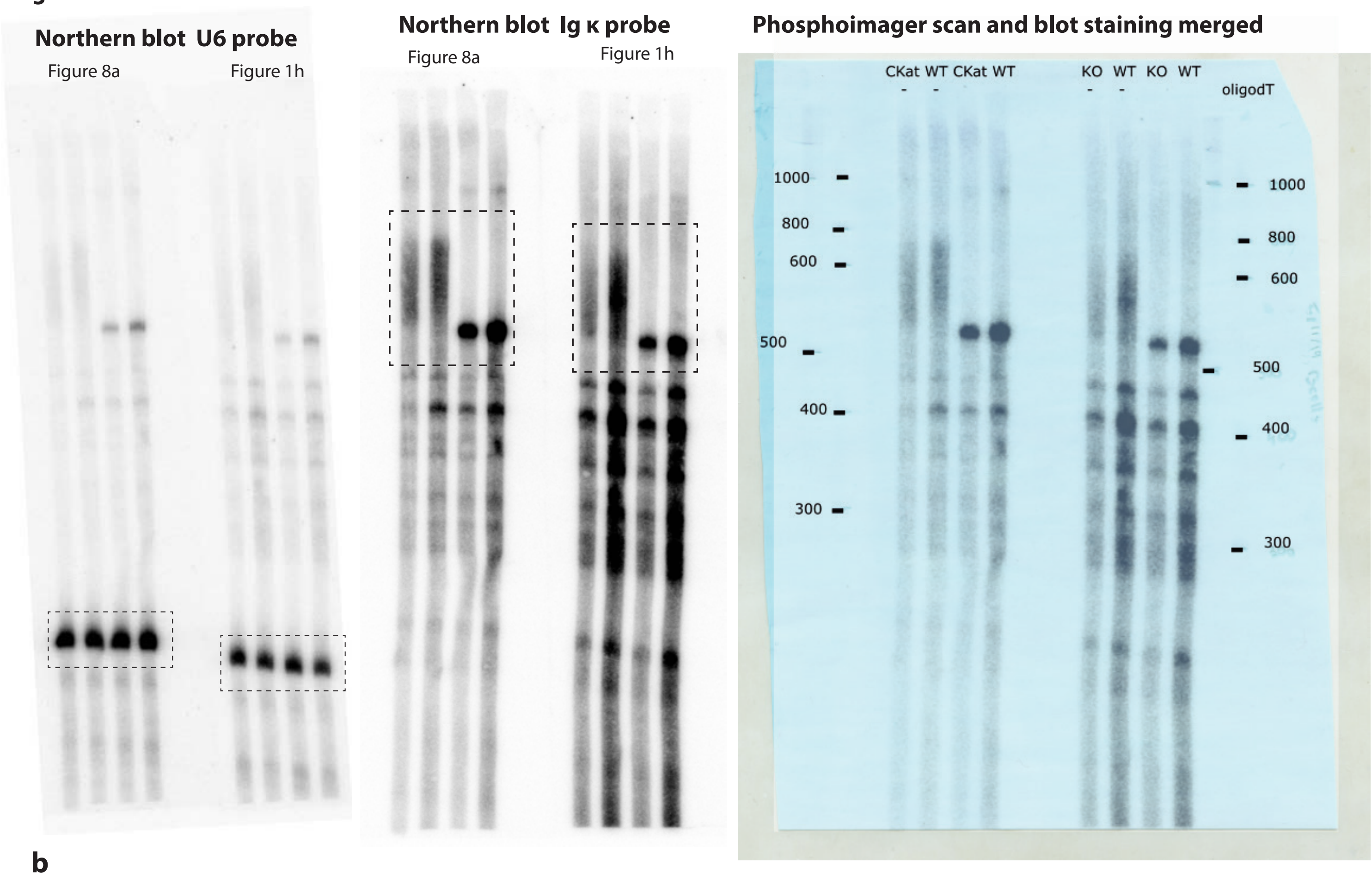

b

Figure 2a

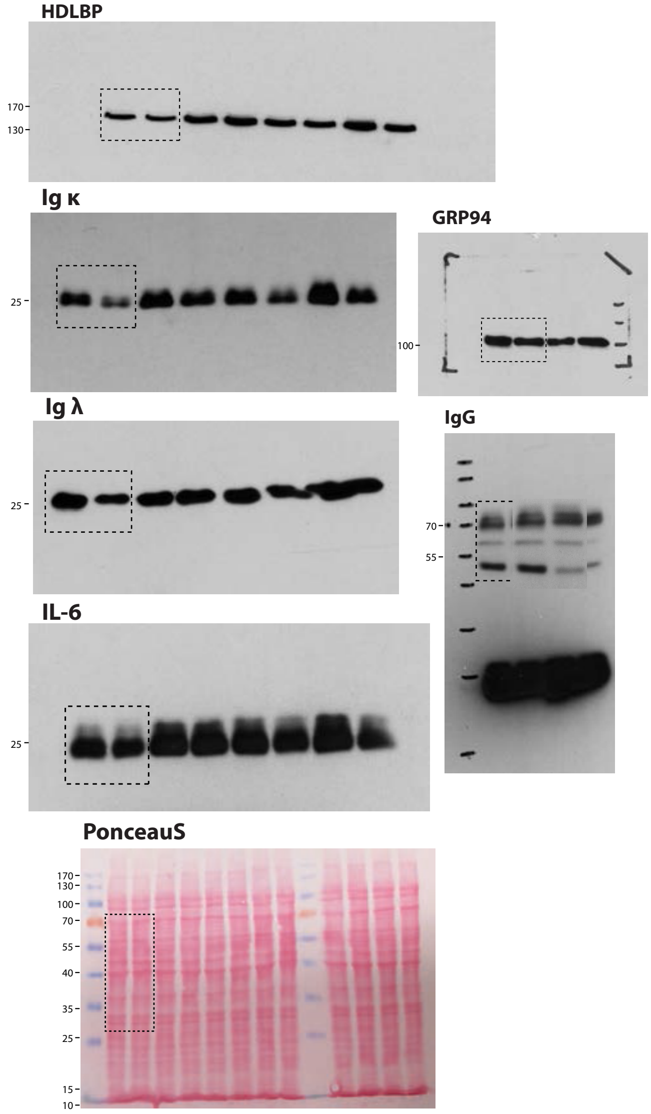

Figure 2b

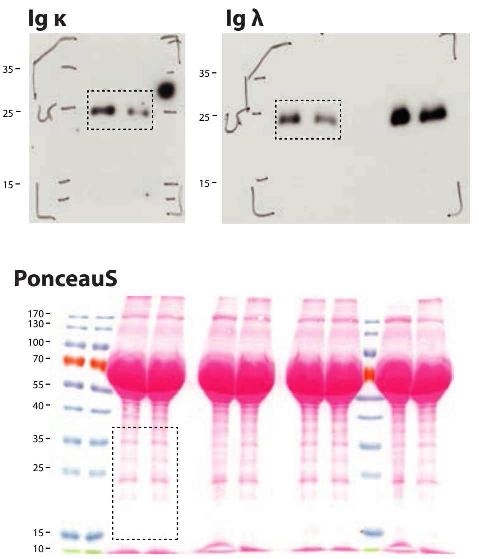

c

Figure 5a

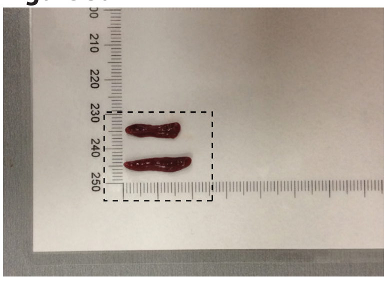

Supplementary Figure 8 related to Supplementary Figure 7a, 7i, 8b, 8c and 8g (continued).

d

Figure 7a

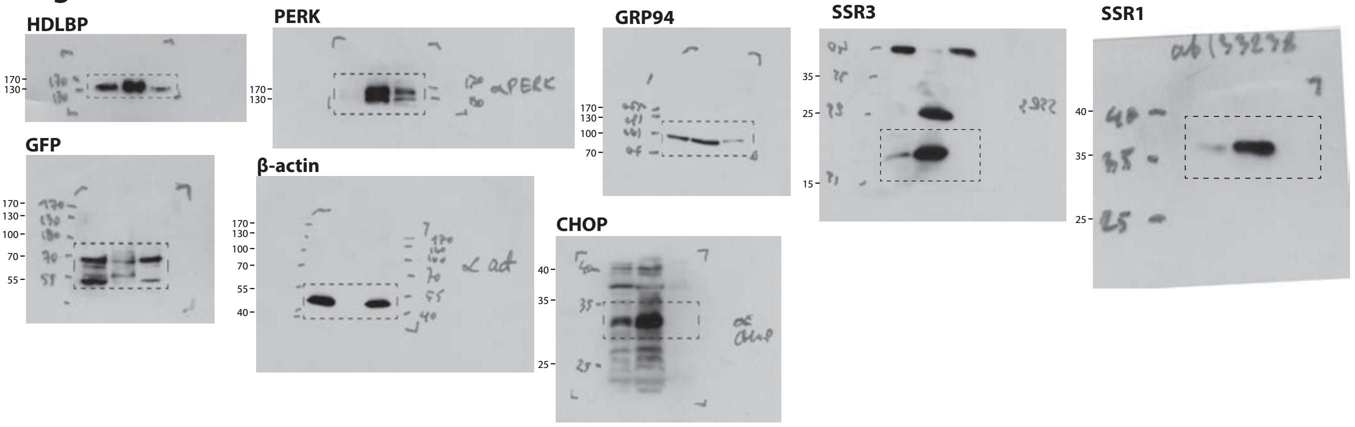

Figure 7i

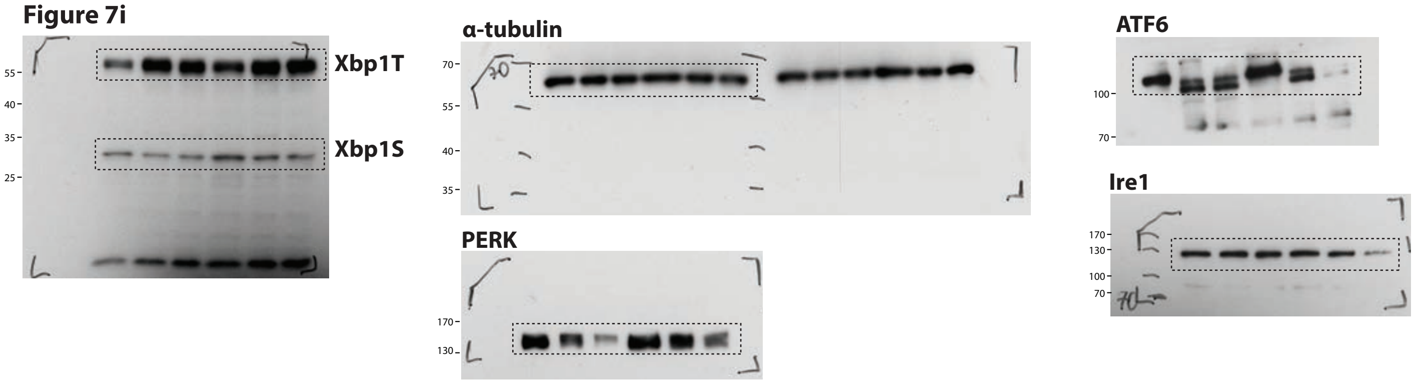

e

Figure 8b

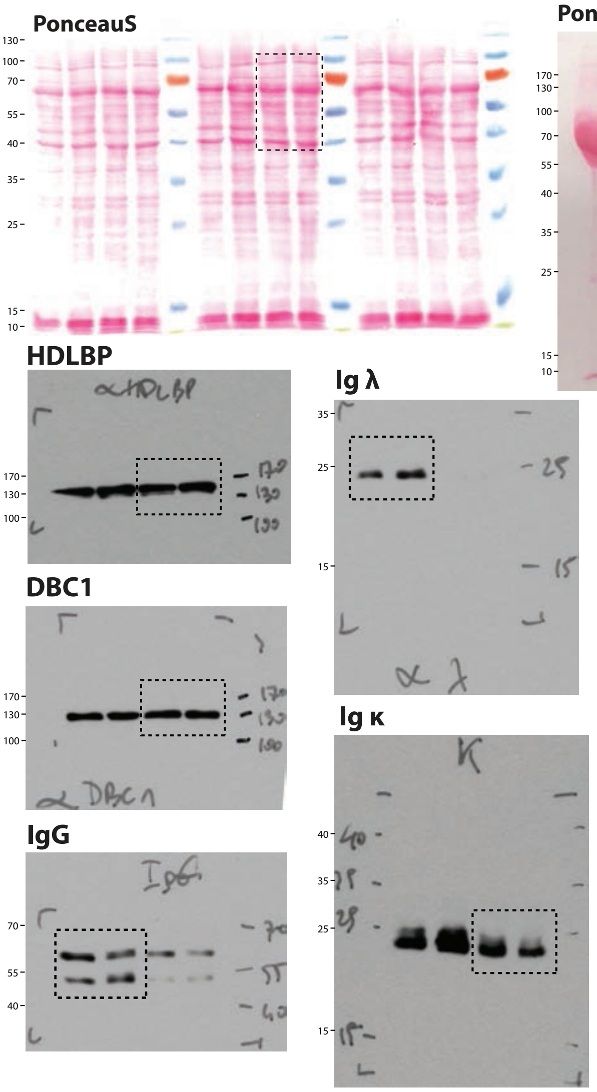

Figure 8c

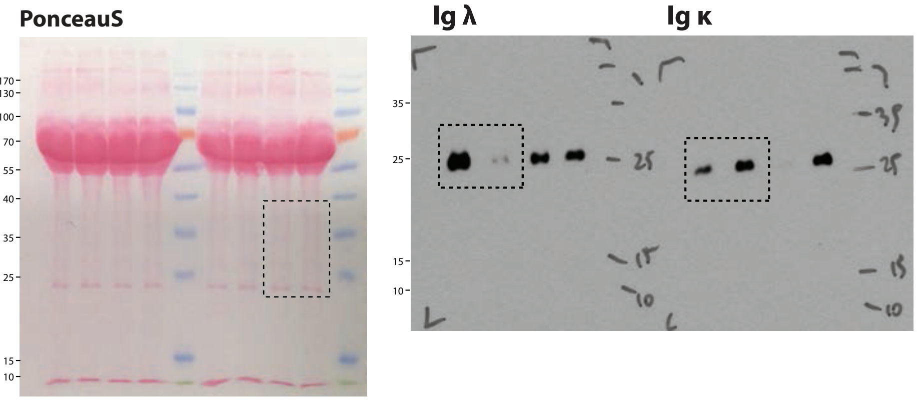

f

Figure 8g

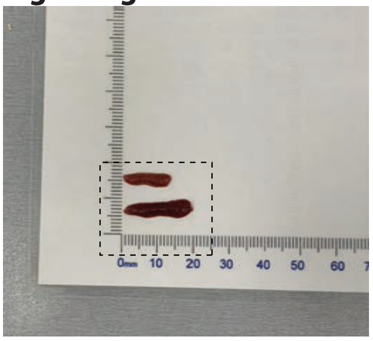

Supplementary Figure 8 related to Supplementary Figure 1a, 1b and 1e (continued).

g

Supplementary Figure 1a

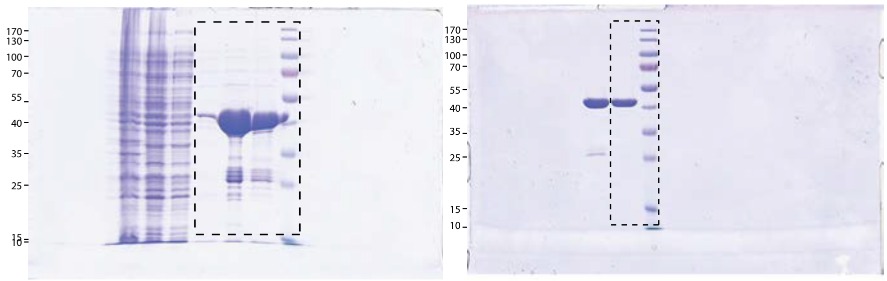

Supplementary Figure 1b

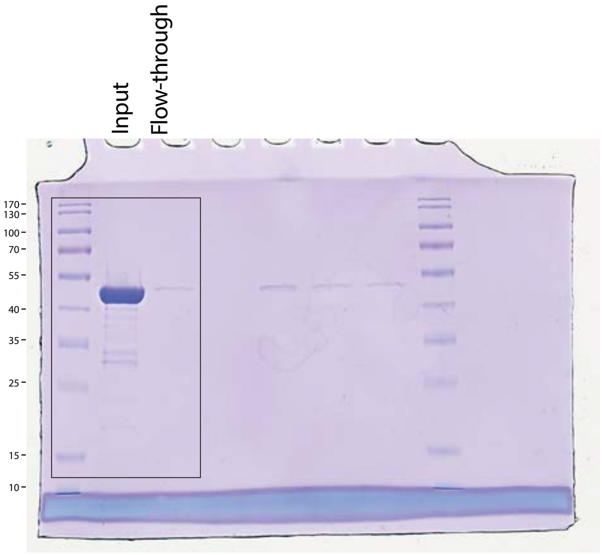

h

Supplementary Figure 1e

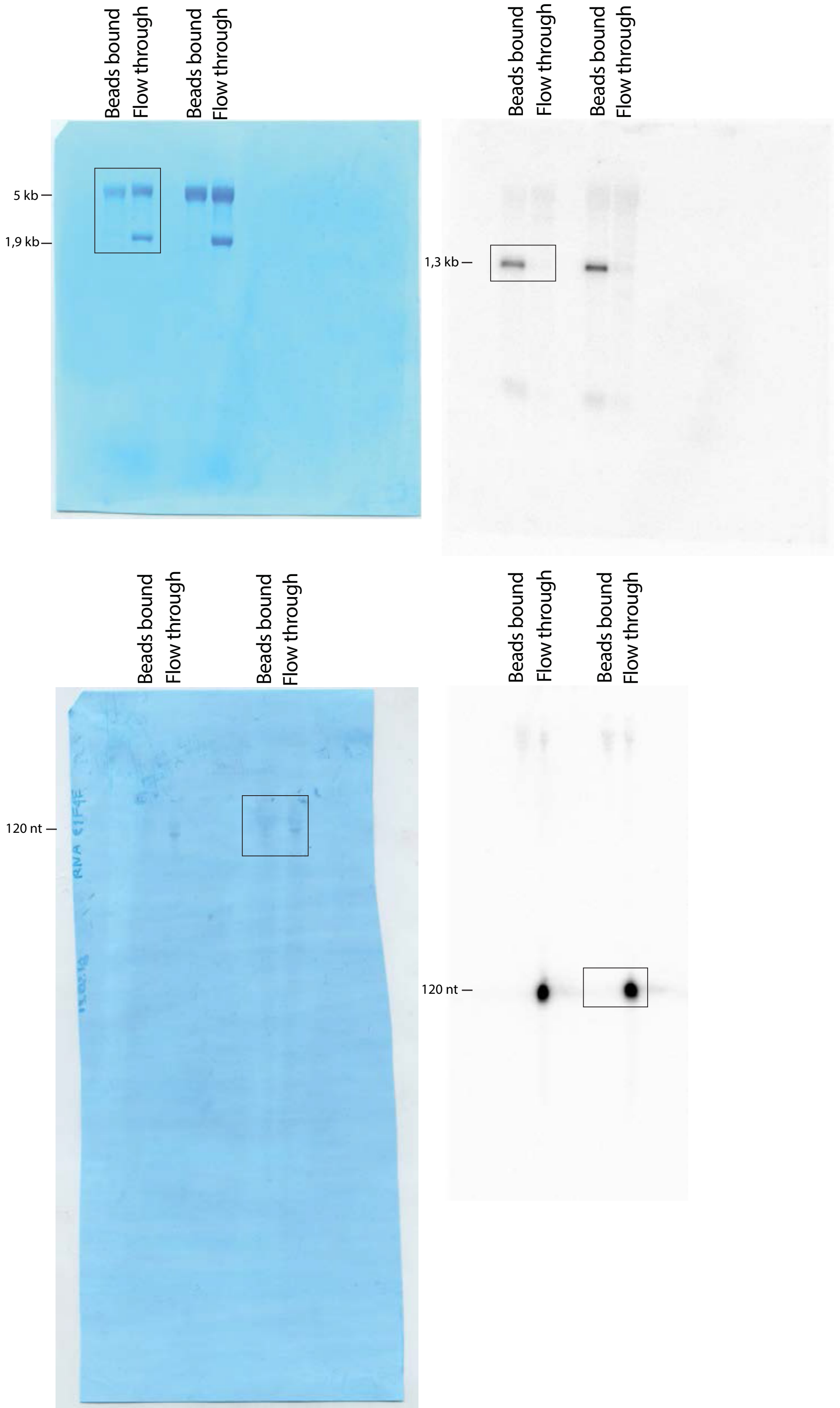

Supplementary Figure 8 related to Supplementary Figure 5a and b (continued).

i

Supplementary Figure 5a and b (secreted proteins).

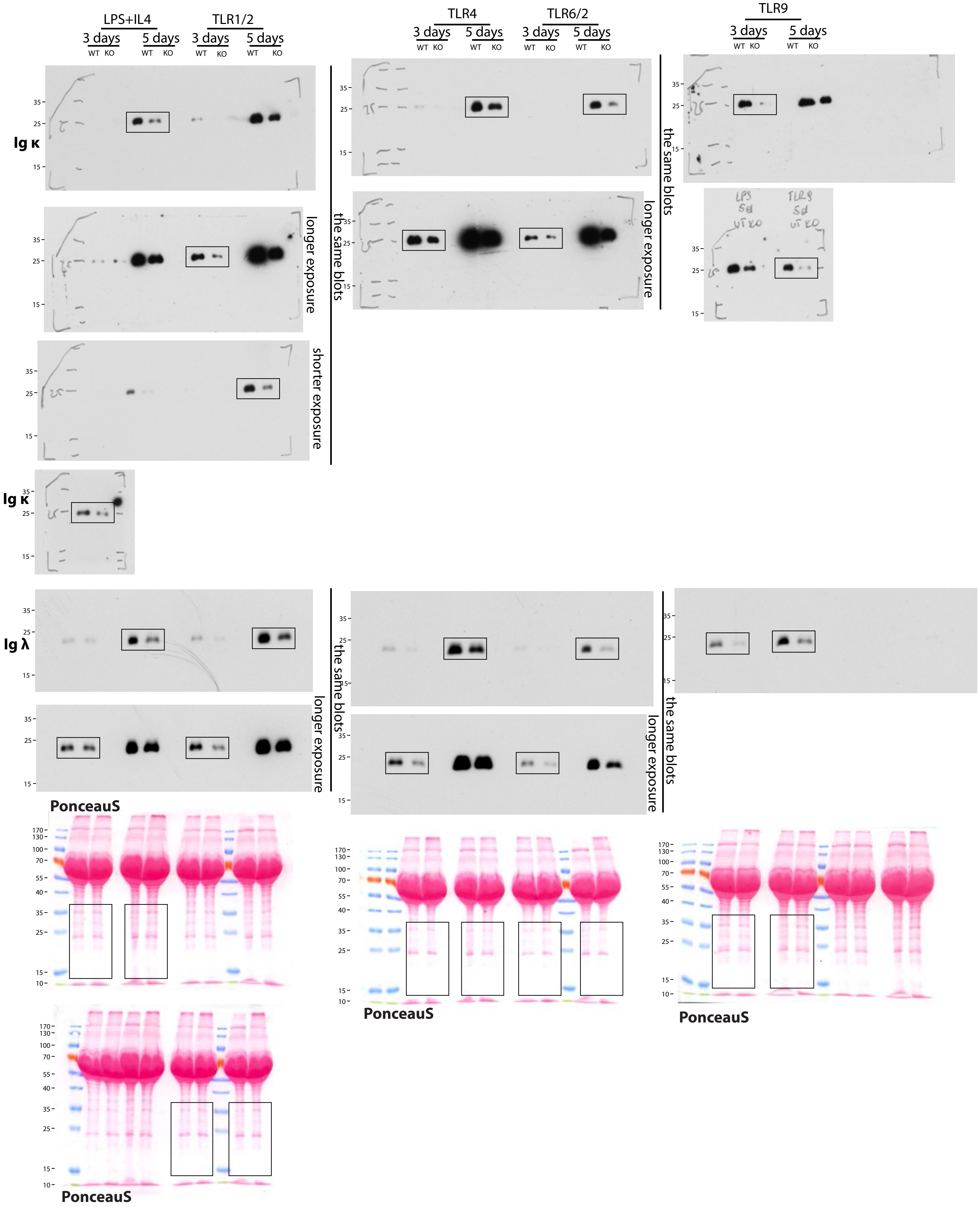

Supplementary Figure 8 related to Supplementary Figure 5a and b (continued).

j

Supplementary Figure 5a and b (intracellular proteins).

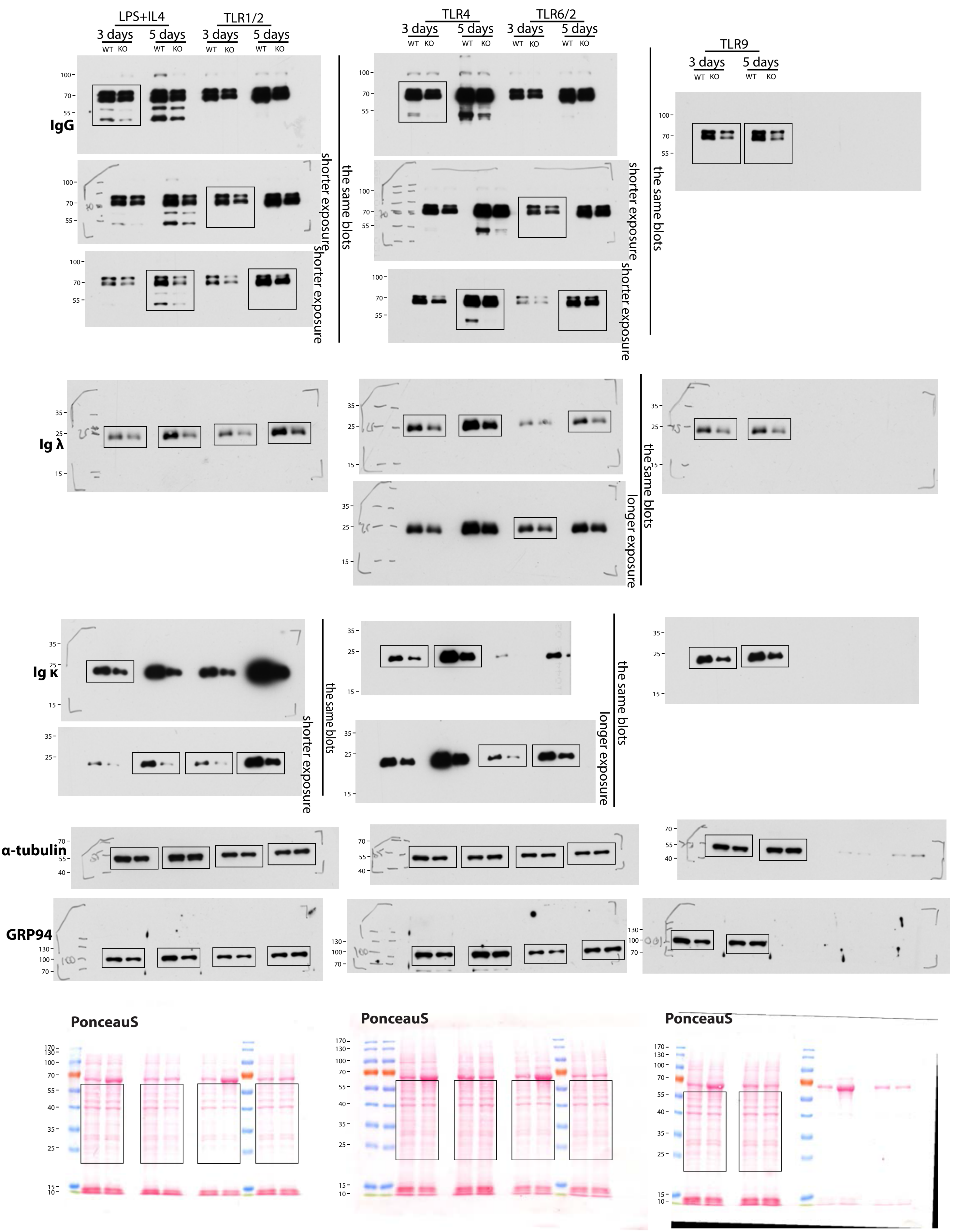

Supplementary Figure 8. Unprocessed scans of blots, gels, and images.

a, Scan of northern blots and blot staining corresponding to Fig. 1h and Fig. 8a.

b, Western blots and membrane Ponceau S staining corresponding to Fig. 2a and Fig. 2b.

c, Spleens photo corresponding to Fig. 5a.

d, Western blots corresponding to Fig. 7a and Fig. 7i.

e, Western blots and membrane Ponceau S staining corresponding to Fig. 8b and Fig. 8c.

f, Spleens photo corresponding to Fig. 8g.

g, Scan of SDS-PAGE gels corresponding to Supplementary Fig. 1a and Supplementary Fig. 1b.

h, Northern blots and methylene blue staining corresponding to Supplementary Fig. 1e.

i, Western blots corresponding to Supplementary Fig. 5a, b (secreted proteins).

j, Western blots corresponding to Supplementary Fig. 5a, b (intracellular proteins).

Supplementary Tables

Supplementary Table 1. Summary of ONT sequencing runs.

Sample	Flowcell	Kit for library preparation	Basecaller	Poly(A) standards added[1]	poly(A) RNA from *S.cerevisiae* added[2]	rRNA depletion step[3]	sequencing reads obtained from a single run	Calibration strand reads[2]	Mapped reads (Mouse reference)	Nanopolish 'PASS' reads
WT_1	R9.4.1 RevC	SQK-RNA001	Guppy 3.3.0	no	no	yes	791983	341596	285413	242314
WT_2	R9.4.1 RevC	SQK-RNA001	Guppy 3.3.0	no	no	yes	570323	236204	221742	175589
WT_3	R9.4.1 RevD	SQK-RNA002	Guppy 3.3.0	yes	yes	no	3356616	0	631521	469603
KO_1	R9.4.1 RevC	SQK-RNA001	Guppy 3.3.0	no	no	yes	492860	178111	202852	177151
KO_2	R9.4.1 RevC	SQK-RNA001	Guppy 3.3.0	no	no	yes	974972	339154	472169	384737
KO_3	R9.4.1 RevD	SQK-RNA002	Guppy 3.3.0	yes	yes	no	2963970	0	717336	510905
							**9150724**	**1095065**	**2531033**	**1960299**

[1] DNA templates for poly(A) standards preparation were a kind gift from prof. Eivind Valen laboratory and were described previously[1].

[2] Instead of the standard calibration strand from the manufacturer kit, we are adding poly(A)+ RNA from *S. cerevisiae*, to assure good quality of sequencing run. *S. cerevisiae* reads are later filtered out by mapping to the yeast reference and may serve as additional control of poly(A) measurements

[3] Although initially RiboZero kit (Illumina) was used to get rid of rRNAs we decided to omit this step, to reduce execution time, simplify and reduce the cost of the procedure. However, we have not noticed any decrease in the reads quality, throughput and reproducibility of sequencing.

Supplementary Table 2. Mice strains used in this study.

Mice strain	Reference
B6CBAF1;B6-TENT5C KO/Tar.<br>All experiments were performed on littermates.	www.crisprmice.eu; Ref. [2]
B6CBAF1;B6-TENT5C Cat (D90N; D92N)/Tar.	This study; www.crisprmice.eu
B6CBAF1;B6-TENT5C$^{GFP/GFP}$/Tar.	This study; www.crisprmice.eu

Supplementary Table 3. List of pathogens detected in *Tent5c* KO and *Tent5c* WT mice.

Pathogen	Method of detection	Results 2018	Results 2017
Ectromelia	Serology	Negative	Negative
EDIM	Serology	Weak positive	Negative
LCMV	Serology	Negative	Negative
Mycoplasma pulmonis	Serology	Negative	Negative
MAV1	Serology	Negative	Negative

MAV2	Serology	Negative	Negative
MHV	Serology	Positive	Positive
MNV	Serology	Positive	Positive
MPV	Serology	Negative	Negative
MVM	Serology	Negative	Negative
PVM	Serology	Negative	Negative
REO3	Serology	Negative	Negative
TMEV	Serology	Negative	Negative
Sendai	Serology	Negative	Negative
Clostridium piliforme	PCR evaluation from feces	Negative	Negative
Citrobacter rodentium	PCR evaluation from feces	Negative	Negative
Corynebacterium kutscheri	PCR evaluation from feces	Negative	Negative
Cryptosporidium spp.	PCR evaluation from feces	Negative	Negative
Helicobacter spp.	PCR evaluation from feces	Positive	Positive
Helicobacter bilis	PCR evaluation from feces	Negative	Negative
Helicobacter ganmani	PCR evaluation from feces	Positive	Positive
Helicobacter hepaticus	PCR evaluation from feces	Negative	Negative
Helicobacter mastomyrinus	PCR evaluation from feces	Negative	Negative
Helicobacter rodentium	PCR evaluation from feces	Negative	Negative
Helicobacter typhlonius	PCR evaluation from feces	Positive	Positive
Pasteurella pneumotropica biotype Jawetz	PCR evaluation from feces	Positive	Positive
Pasteurella pneumotropica biotype Heyl	PCR evaluation from feces	Positive	Positive
Salmonella spp.	PCR evaluation from feces	Negative	Negative
Streptobacillus moniliformis	PCR evaluation from feces	Negative	Negative
Streptococcus pneumoniae	PCR evaluation from feces	Negative	Negative
Streptococcus sp. beta hemolytic Group A	PCR evaluation from feces	Negative	Negative
Streptococcus sp. beta hemolytic Group B	PCR evaluation from feces	Negative	Negative
Streptococcus sp. beta hemolytic Group C	PCR evaluation from feces	Negative	Negative
Streptococcus sp. beta hemolytic Group G	PCR evaluation from feces	Negative	Negative
Giardia muris	PCR evaluation from feces	Negative	Negative
Spironucleus muris	PCR evaluation from feces	Positive	Positive
Aspiculuris tetraptera	PCR evaluation from feces	Positive	Positive
Syphacia muris	PCR evaluation from feces	Negative	Negative
Syphacia obvelata	PCR evaluation from feces	Positive	Positive

Myocoptes	Pelt swap	Positive	Positive
Radfordia/Myobia	Pelt swap	Positive	Positive

Supplementary Table 4. Primers used for generation of *Tent5c*-GFP *knock-in* mice and genotyping.

Primer name	Sequence
mTENT5C_GFP_gRNA_F	GAAATTAATACGACTCACTATAGGGAGGTCTTCAGGTTAGTTACGTTTTAGAGCTAGAAATAGCAAGTTAAAATAAGGC
Universal_gRNA_rev	CTTCAGAACCACTTCTCGGA
mTent5C_TOPO-LF_1f	CCACTAGTAACGGCCGCCAGTGTGCTGGAATTCTCGAGGAGATAACCCTGAAGGACA
mTent5C_LF-TEV_1r	ACCATGATATCACCCTGAAAATACAAATTCTCGTTACAGGGCAGCCATG
mTent5C_eGFP-RF_1f	ATCACTCTCGGCATGGACGAGCTGTACAAGTAACCTGAAGACCTGAGGG
mTent5C_RF-TOPO_1r	CGGCCGCCAGTGTGATGGATATCTGCAGAATTCTTTTCATACTGGGAGTGACG
TEV_1f	GAGAATTTGTATTTTCAGGGTGA
mCherry_eGFP_1r	TTACTTGTACAGCTCGTCCA
mTent5C_GFP_short_1F	TACATTGCGCACCCTCCAATTACC
mTent5C_GFP_short_1R	TACCTGAGAGCCCCTGCCCT
mTent5C_GFP_seq1F	CTTCAGAACCACTTCTCGGA
mTent5C_GFP_seq1R	AGAAGTCACGCCTCCTATTG
Tent5C_catODN	AGCCGGCCACGTTTTGGTCAAAGACAACGGCTTGGGTTGCAAAaATCTGaATCTGATCTTTCACGTGGCTCTCCCCACAGAGGCG
Fam46C_seq2F	AGGTCCTGACTGAGGTCGTG
Fam46C_seq2R	TTCCTCAAAATCCCCGTACA

Supplementary Table 5. List of primers used in this study.

Primer	Sequence	Species	Reference	Application
Igkc F	GTGCCTCAGTCGTGTGCTTC	Mouse	This study	qPCR
Igkc R	TGCTGCTCATGCTGTAGGTG	Mouse	This study	qPCR
Iglc F	AGACTTCGCCATCAGTCACC	Mouse	This study	qPCR

Iglc R	CCAGTCCACTGTCACCACAC	Mouse	This study	qPCR
Ighm F	AACAGAGATCTGCATGTGCC	Mouse	This study	qPCR
Ighm R	TTCGTGGCCTCGCAGATGAG	Mouse	This study	qPCR
Jchain F	GACGATGGTGTTCCTGAGAC	Mouse	This study	qPCR
Jchain R	CAAGCTAGTCAGGGTAGCAAG	Mouse	This study	qPCR
Gapdh F	AAGGGCTCATGACCACAGTC	Mouse	Ref. [3]	qPCR
Gapdh R	GGATGACCTTGCCCACAG	Mouse	Ref. [3]	qPCR
Tent5c F	CAGTCACCTCCTCTTCCAACG	Mouse	This study	qPCR
Tent5c R	AACCTGATCCCAGTTGAGCAC	Mouse	This study	qPCR
Pabpc1 F	TGCAGAGGATGGCAAGTGTACG	Mouse	Ref. [4]	qPCR
Pabpc1 R	GCTAGGAGGATAGTATGCAGC	Mouse	Ref. [4]	qPCR
Xbp1 T F	TGGCCGGGTCTGCTGAGTCCG	Mouse	Ref. [5]	qPCR
Xbp1 R1	GTGTCAGAGTCCATGGGA	Mouse	This study	qPCR
Xbp1 S F	CTGAGTCCGAATCAGGTGCAG	Mouse	Ref. [5]	qPCR
Xbp1 US F	CAGCACTCAGACTATGTGCA	Mouse	Ref. [5]	qPCR
Xbp1 R2	GTCCAACTTGTCCAGAATGCC	Mouse	This study	qPCR
Perk F	TGTCTTGGTTGGGTCTGATG	Mouse	This study	qPCR
Perk R	ACCGTTATCGTATGGATACTGG	Mouse	This study	qPCR
Chop F	CTGCCTTTCACCTTGGAGAC	Mouse	Ref. [6]	qPCR
Chop R	CGTTTCCTGGGGATGAGAT	Mouse	Ref. [6]	qPCR
Grp94 F	TCAAATCGAACACGGCTTGC	Mouse	This study	qPCR
Grp94 R	CCATGAAGTAGATTTTGTCC	Mouse	This study	qPCR
Ero1lB F	AAGTACTCGCAAGCAGCAAACAGC	Mouse	Ref. [7]	qPCR
Ero1lB R	TATCTCGCCCAGTCAATGAACGCT	Mouse	Ref. [7]	qPCR
Ire1 F	GCCGAAGTTCAGATGGAATC	Mouse	Ref. [8]	qPCR
Ire1 R	ATCAGCAAAGGCCGATGA	Mouse	Ref. [8]	qPCR
5S rRNA F	CATACCACCCTGAACGCG	Human	This study	qPCR
5S rRNA R	CTACAGCACCCGGTATTCCC	Human	This study	qPCR
18S rRNA F	GAGAAACGGCTACCACATCCAA	Human	Ref. [9]	qPCR
18S rRNA R	CCAATTACAGGGCCTCGAAAGA	Human	Ref. [9]	qPCR
5.8S rRNA F	GGTGGATCACTCGGCTCGT	Human	Ref. [10]	qPCR
5.8S rRNA R	CCGCAAGTGCGTTCGAAGTG	Human	Ref. [10]	qPCR
28S rRNA F	GGGTGGTAAACTCCATCTAAGG	Human	Ref. [11]	qPCR

28S rRNA R	GCCCTCTTGAACTCTCTCTTC	Human	Ref. [11]	qPCR
GAPDH F	ATCAAGAAGGTGGTGAAGCA	Human	Ref. [12]	qPCR
GAPDH R	CATACCAGGAAATGAGCTTG	Human	Ref. [12]	qPCR
ACTB F	GCATGGGTCAGAAGGATTCC	Human	Ref. [13]	qPCR
ACTB R	CCACACGCAGCTCATTGTAG	Human	Ref. [13]	qPCR
Igkc F	CTGTCAGTCTTGGAGATCAAG	Mouse	This study	Northern blot probe
Igkc R	CCGAACGTGTACGGAACATG	Mouse	This study	Northern blot probe
U6	AACGCTTCACGAATTTGCGT	Mouse	This study	Northern blot probe
IgK_RNaseH	GATGGATACAGTTGGTGCAGC	Mouse	This study	RNase H assay

Supplementary Table 6. List of antibodies and recombinant proteins used for WB, FACS or cells activation.

	Antibody or recombinant protein	Source	Identifier	Lot number
1	Rabbit monoclonal anti-Ire1; Clone: 14C10	CST	Cat# 3294<br>RRID:AB_823545	Lot# 11 Ref: 10/2018
2	Rabbit monoclonal anti-ATF6; Clone D4Z8V	CST	Cat# 65880<br>RRID:AB_2799696	Lot# 2 Ref: 10/2018
3	Rabbit monoclonal anti-PERK; Clone C33E10	CST	Cat# 3192<br>RRID:AB_2095847	Lot# 10 Ref: 11/2018
4	Mouse monoclonal anti-α-tubulin; Clone DM1A	Millipore	Cat# CP06<br>RRID:AB_2617116	Lot# 2972830
5	Rabbit monoclonal anti-Xbp1; Clone EPR22004	Abcam	Cat# ab220783	Lot# GR3238989-4
6	F(ab')2-Goat anti-Mouse IgM (Mu chain); Polyclonal	Thermo Fisher Scientific	Cat# 16-5092-85<br>RRID:AB_2573088	Lot# 2001782
7	Mouse monoclonal anti-IL6; Clone 10E5	SCBT	Cat# sc-57315<br>RRID:AB_2127596	Lot# F2717
8	m-IgGλ BP-HRP; Purified recombinant mouse protein conjugated to HRP	SCBT	Cat# sc-516132	Lot# L0617
9	m-IgGκ BP-HRP; Purified recombinant mouse protein conjugated to HRP	SCBT	Cat# sc-516102<br>RRID:AB_2687626	Lot# K2818
10	Rabbit polyclonal anti-GRP94; Clone H-212	SCBT	Cat# sc-11402<br>RRID:AB_2119050	Lot# C1616
11	Rabbit anti-GFP; Polyclonal	ChromoTek	Cat# PABG1-100<br>RRID:AB_2749857	Lot# 70828032AB

12	Rabbit anti-HDLBP; Polyclonal	Bethyl	Cat# A303-971A, RRID:AB_2620320	Lot# A303-971A-1
13	Rabbit monoclonal anti-CHOP; Clone D46F1	CST	Cat# 5554, RRID:AB_10694399	Lot# 5 Ref: 11/2018
14	Rabbit monoclonal anti-TRAPα; Clone EPR5603	Abcam	Cat# ab133238, RRID:AB_11157579	Batch finished
15	Rabbit polyclonal Anti-SSR3; Polyclonal	Abcam	Cat# ab190936	Batch finished
16	Mouse monoclonal anti-β-actin; Clone C4	Millipore	Cat# MAB1501, RRID:AB_2223041	Batch finished
17	BUV395 Rat monoclonal anti-CD19; Clone 1D3	BD Biosciences	Cat# 563557 RRID:AB_2722495	Lot# 9063653
18	AF700 Rat monoclonal anti-CD19; Clone 1D3	BD Biosciences	Cat# 557958 RRID:AB_396958	Lot# 7201927
19	BV421 Rat monoclonal anti-CD43; Clone S7	BD Biosciences	Cat# 562958 RRID:AB_2665409	Lot# 8201932
20	BV421 Rat monoclonal anti-CD23; Clone B3B4	BD Biosciences	Cat# 562929 RRID:AB_2737898	Lot# 8194818
21	FITC Rat monoclonal anti-IgM; Clone II/41	BD Biosciences	Cat# 553437 RRID:AB_394857	Lot# 6275818
22	PE Mouse monoclonal anti-CD249 (Ly-51); Clone BP-1	BD Biosciences	Cat# 553735 RRID:AB_395018	Lot# 8096737
23	PE Rat monoclonal anti-CD93 (Early B Lineage); Clone AA4.1	BD Biosciences	Cat# 558039 RRID:AB_397003	Lot# 6259562
24	PE Rat monoclonal anti-IgA; Clone 11-44-2	Thermo Fisher Scientific	Cat# 12-5994-81 RRID:AB_466115	Lot# 2090626
25	PE Rat monoclonal anti-CD138; Clone 281-2	BD Biosciences	Cat# 553714 RRID:AB_395000	Lot# 7096908
26	BV605 Rat monoclonal anti-CD138; Clone 281-2	BD Biosciences	Cat# 563147 RRID:AB_2721029	Lot# 9049652
27	BV605 Rat monoclonal anti-IgD; Clone 11-26c.2a	BD Biosciences	Cat# 563003 RRID:AB_2737944	Lot# 8234705
28	AF647 Goat anti-IgG1; Polyclonal	Thermo Fisher Scientific	Cat# A-21240 RRID:AB_ 2535809	Lot# 2092295
29	APC Rat monoclonal anti-CD45R/B220; Clone RA3-6B2	BD Biosciences	Cat# 553092 RRID:AB_398531	Lot# 8086734
30	PerCPCy5.5 Rat monoclonal anti-CD45R/B220; Clone RA3-6B2	BD Biosciences	Cat# 552771 RRID:AB_394457	Lot# 7326610
31	PerCPCy5.5 Rat monoclonal anti-CD24; Clone M1/69	BD Biosciences	Cat# 562360 RRID:AB_11151895	Lot# 8116606

32	PerCPCy5.5 Rat monoclonal anti-CD21/CD35; Clone 7G6	BD Biosciences	Cat# 562797 RRID:AB_2737802	Lot# 8110782
33	Chicken anti-GFP; Polyclonal	Abcam	Cat# ab13970 RRID:AB_300798	Lot# GR3190550-9
34	IL-4 (recombinant protein)	Peprotech	Cat# 214-14	Lot# 081449 G1116
35	megaCD40L (recombinant protein)	Enzo Lifesciences	Cat# ALX-522-120-C010	Lot# 11231606
36	Goat Anti-Mouse IgG, H&L Chain Specific Peroxidase Conjugate	Millipore	Cat# 401215 RRID:AB_10682749	Lot# D0016470
37	Goat Anti-Rabbit IgG, H&L Chain Antibody, Peroxidase Conjugated	Millipore	Cat # 401393 RRID:AB_10683386	Lot# 2854069
38	Rat anti-Mouse CD16/32, Clone 2.4G2	BD Biosciences	Cat#553142 RRID:AB_394657	Lot# 8179557
39	Alexa 488 Goat anti-Chicken, Polyclonal	Thermo Fisher Scientific	Cat# A-11039 RRID:AB_2534096	Batch finished

Supplementary Table 7. List of software and algorithms used in this study.

Software and Algorithms	Source	Identifier
Prism 6 for Windows	GraphPad Prism	RRID: SCR_002798
FlowJo v10	FlowJo, LLC	RRID: SCR_008520
MultiGauge v 3.0	FujiFilm (discontinued)	RRID:SCR_014299
ImageJ 1.52p	https://imagej.nih.gov/ij/	RRID:SCR_003070
R Project for Statistical Computing v. 3.6.0	R Foundation for Statistical Computing, Vienna, Austria	RRID:SCR_001905
Cutadapt v. 1.18	Ref. [14] http://journal.embnet.org/index.php/embnetjournal/article/view/200	RRID:SCR_011841
Subread v. 1.6.3	Ref. [15] http://subread.sourceforge.net/	RRID:SCR_009803
Guppy 3.3.0	Oxford Nanopore Technologies	N/A
DESeq2 v. 1.22	Ref. [16]	RRID:SCR_015687
STAR split read aligner v. 2.6.1a	Ref. [17] https://github.com/alexdobin/STAR	RRID:SCR_015899
Minimap 2.16	Ref. [18] https://github.com/lh3/minimap2	N/A
Nanopolish 0.11.1	Ref. [19] https://github.com/jts/nanopolish	RRID:SCR_016157

NanoTail R	This study;<br>DOI: 10.5281/zenodo.3227971<br>https://github.com/pbrigf-ibb/nanotail	N/A
Mutation Surveyor 4.0	SoftGenetics<br>http://www.softgenetics.com/mutationSurveyor.php	RRID:SCR_001247
FACS Diva Software v. 8.0.1	Becton Dickinson	N/A
MinKNOW 19.10.1	Oxford Nanopore Technologies	N/A
Platinum software V6	Helena-Biosciences	N/A

Supplementary References

1. Krause, M. *et al.* tailfindr: alignment-free poly(A) length measurement for Oxford Nanopore RNA and DNA sequencing. *RNA* **25**, 1229-1241 (2019).
2. Mroczek, S. *et al.* The non-canonical poly(A) polymerase FAM46C acts as an onco-suppressor in multiple myeloma. *Nat. Commun.* **8**, 619 (2017).
3. Kakiuchi-Kiyota, S. *et al.* Evaluation of direct and indirect effects of the PPARgamma agonist troglitazone on mouse endothelial cell proliferation. *Toxicol. Pathol.* **39**, 1032-1045 (2011).
4. Chorghade, S. *et al.* Poly(A) tail length regulates PABPC1 expression to tune translation in the heart. *eLife* **6**, e24139 (2017).
5. Oslowski, C.M. & Urano, F. Measuring ER stress and the unfolded protein response using mammalian tissue culture system. *Methods Enzymol.* **490**, 71-92 (2011).
6. Serrano, R.L., Yu, W. & Terkeltaub, R. Mono-allelic and bi-allelic ENPP1 deficiency promote post-injury neointimal hyperplasia associated with increased C/EBP homologous protein expression. *Atherosclerosis* **233**, 493-502 (2014).
7. Aragon, I.V., Barrington, R.A., Jackowski, S., Mori, K. & Brewer, J.W. The specialized unfolded protein response of B lymphocytes: ATF6alpha-independent development of antibody-secreting B cells. *Mol. Immunol.* **51**, 347-355 (2012).
8. Tsuru, A., Imai, Y., Saito, M. & Kohno, K. Novel mechanism of enhancing IRE1alpha-XBP1 signalling via the PERK-ATF4 pathway. *Sci. Rep.* **6**, 24217 (2016).
9. Bauer, A.K. *et al.* Transcriptomic analysis of pathways regulated by toll-like receptor 4 in a murine model of chronic pulmonary inflammation and carcinogenesis. *Mol. Cancer* **8**, 107 (2009).
10. Loughlin, F.E. *et al.* The Solution Structure of FUS Bound to RNA Reveals a Bipartite Mode of RNA Recognition with Both Sequence and Shape Specificity. *Mol. Cell* **73**, 490-504 (2019).
11. Hald, O.H. *et al.* Inhibitors of ribosome biogenesis repress the growth of MYCN-amplified neuroblastoma. *Oncogene* **38**, 2800-2813 (2019).
12. Kobylecki, K., Drazkowska, K., Kulinski, T.M., Dziembowski, A. & Tomecki, R. Elimination of 01/A'-A0 pre-rRNA processing by-product in human cells involves cooperative action of two nuclear exosome-associated nucleases: RRP6 and DIS3. *RNA* **24**, 1677-1692 (2018).

13. Eaton, J.D. *et al.* Xrn2 accelerates termination by RNA polymerase II, which is underpinned by CPSF73 activity. *Genes Dev.* **32**, 127-139 (2018).
14. Martin, M. Cutadapt removes adapter sequences from high-throughput sequencing reads. *EMBnet J.* **17**, 3 (2011).
15. Liao, Y., Smyth, G.K. & Shi, W. featureCounts: an efficient general purpose program for assigning sequence reads to genomic features. *Bioinformatics* **30**, 923-930 (2014).
16. Love, M.I., Huber, W. & Anders, S. Moderated estimation of fold change and dispersion for RNA-seq data with DESeq2. *Genome Biol.* **15**, 550 (2014).
17. Dobin, A. *et al.* STAR: ultrafast universal RNA-seq aligner. *Bioinformatics* **29**, 15-21 (2013).
18. Li, H. Minimap2: pairwise alignment for nucleotide sequences. *Bioinformatics* **34**, 3094-3100 (2018).
19. Workman, R.E. *et al.* Nanopore native RNA sequencing of a human poly(A) transcriptome. *Nat. Methods* **16**, 1297–1305 (2019).
