# Supplementary material for: Immunoglobulin expression and the humoral immune response is regulated by the non-canonical poly(A) polymerase TENT5C
Source: Nat Commun. 2020 Apr 27;11:2032. doi: 10.1038/s41467-020-15835-3 (PMC7184606; doi:10.1038/s41467-020-15835-3)
Supplement: Supplementary file 3 — Description of Additional Supplementary Files [file 41467_2020_15835_MOESM3_ESM.docx]

**Description of Additional Supplementary Files**

**File name:** Supplementary Dataset 1

**Description:** Results of differential adenylation analysis on data from Nanopore direct RNA sequencing of RNA isolated from B cells of WT and Tent5c KO mice activated for 7 days.

**File name:** Supplementary Dataset 2

**Description:** Results of differential expression analysis on data from Illumina sequencing of RNA isolated from B cells of WT and Tent5c KO mice activated for 7 days.

**File name:** Supplementary Dataset 3

**Description:** Mass spectrometry data from Tent5c-GFP immunoprecipitations from activated B cells.
